# Using oral and parenteral formulation of AWaRe antibiotics as a proxy estimate of community and hospital healthcare sector use

**DOI:** 10.1093/jacamr/dlag057

**Published:** 2026-04-30

**Authors:** Cherry Lim, Ines Pauwels, Hoa Q Nguyen, Will Cuningham, Mike Thorn, Aislinn Cook, Myo Maung Maung Swe, Ben S Cooper, Benedikt Huttner, Yingfen Hsia, Catrin E Moore, Erika Vlieghe, Koen B Pouwels, Ann Versporten, Michael Sharland

**Affiliations:** Centre for Tropical Medicine and Global Health, Nuffield Department of Medicine, University of Oxford, Oxford, UK; Mahidol Oxford Tropical Medicine Research Unit, Faculty of Tropical Medicine, Mahidol University, Bangkok, Thailand; Global Health Institute, Department of Family Medicine and Population Health, Faculty of Medicine and Health Sciences, University of Antwerp, Antwerp, Belgium; Laboratory of Medical Microbiology, Vaccine & Infectious Disease Institute, Faculty of Medicine and Health Science, University of Antwerp, Antwerp, Belgium; School of Pharmacy, Queen's University Belfast, Belfast, UK; City St. George's, University of London, London SW17 0RE, UK; City St. George's, University of London, London SW17 0RE, UK; City St. George's, University of London, London SW17 0RE, UK; Nuffield Department of Population Health, University of Oxford, Oxford, UK; Centre for Tropical Medicine and Global Health, Nuffield Department of Medicine, University of Oxford, Oxford, UK; Centre for Tropical Medicine and Global Health, Nuffield Department of Medicine, University of Oxford, Oxford, UK; Mahidol Oxford Tropical Medicine Research Unit, Faculty of Tropical Medicine, Mahidol University, Bangkok, Thailand; Department of Antimicrobial Resistance, Health Systems Division, World Health Organization, Avenue Appia 20, Geneva 1211, Switzerland; City St. George's, University of London, London SW17 0RE, UK; City St. George's, University of London, London SW17 0RE, UK; Global Health Institute, Department of Family Medicine and Population Health, Faculty of Medicine and Health Sciences, University of Antwerp, Antwerp, Belgium; Department of General Internal Medicine, Infectious Diseases and Tropical Medicine, University Hospital Antwerp, Antwerp, Belgium; Nuffield Department of Primary Care Health Sciences, University of Oxford, Oxford, UK; Global Health Institute, Department of Family Medicine and Population Health, Faculty of Medicine and Health Sciences, University of Antwerp, Antwerp, Belgium; Laboratory of Medical Microbiology, Vaccine & Infectious Disease Institute, Faculty of Medicine and Health Science, University of Antwerp, Antwerp, Belgium; City St. George's, University of London, London SW17 0RE, UK

## Abstract

**Background:**

Benchmarking antibiotic use across different healthcare sectors is crucial to improve use and implement the United Nations General Assembly 70% Access target. Many countries only have available aggregate sales data, which do not have sector-specific usage information. The objective of this study is to estimate the proportion of oral and parenteral antibiotic use across different healthcare sectors.

**Materials and methods:**

We used IQVIA MIDAS^®^ Quarterly Sales data and Global Point Prevalence Survey (Global-PPS) hospital healthcare data from eight countries, including Belgium, Canada, China, Netherlands, Philippines, Saudi Arabia, Singapore and the UK, in 2019. Our analysis focused on Access and Watch antibiotics. In the main analysis, we assumed that all parenteral antibiotics were used exclusively in hospital healthcare settings, an assumption we then relaxed through sensitivity analyses. The observed ratios of oral-to-parenteral antibiotics in the patient-level Global-PPS data were calculated, by dividing the volume of oral antibiotic use by that of parenteral antibiotic use, and then this calculated ratio was multiplied by the IQVIA MIDAS sales data to estimate oral antibiotic use outside of the hospital healthcare sector.

**Results:**

The ratios of oral-to-parenteral use among hospital healthcare sectors in the Global-PPS data ranged between 0.05 [95% credible interval (CrI): 0.03–0.09] and 1.01 (95%CrI: 0.56–1.80) in the main analysis. We estimated that overall, <7% of national oral antibiotics were used by hospital healthcare sectors, assuming exclusive parenteral use in hospital healthcare settings in the main analysis, and <9% when assuming 90% of parenteral use was hospital healthcare in the sensitivity analyses.

**Conclusion:**

Our results indicate that where patient-level data are unavailable, alternative sources, such as antibiotic procurement and supply data including routes of administration, can reasonably estimate sector-specific national use.

## Introduction

Antimicrobial resistance (AMR) is a global health threat. It is estimated that the number of deaths due to AMR in 2021 ranged from 1.14 million [95% uncertainty interval (UI): 1.00–1.28] to 4.91 million (95% UI: 4.23–5.19) globally.^1^ Recognizing the important roles of inappropriate antibiotic use in driving the emergence and spread of AMR,^2^ the 79th UN General Assembly set a target for 70% of global human antibiotic use to come from the Access group of the World Health Organization’s (WHO) AWaRe (Access, Watch, Reserve) classification of antibiotics, setting a measurable target for improving antibiotic use.^3^ The development and monitoring of antibiotic use targets at a country level necessitates robust data. Initiatives, such as the Global Point Prevalence Survey of Antimicrobial Consumption and Resistance (Global-PPS),^4^ have contributed to bridging the gap in hospital antibiotic use data, but there are very limited data on primary-care antibiotic use to date. The IQVIA MIDAS Quarterly Sales data,^5^ provides useful data on national antibiotic sales trends, but only for 77 countries.^6^ The WHO launched the Global Antimicrobial Resistance and Use Surveillance System (GLASS) to support the Global Action Plan to understand antibiotic use and tackle AMR.^7,8^ The WHO GLASS-antimicrobial use (AMU) report on antibiotic data in 2022 suggested oral formulation of antibiotics accounts for >90% of the total use in most countries.^7^ Most countries only have access to internal import trade data, which provides a broad overview of national antibiotic use, but does not include the healthcare sector-specific use details (i.e. usage in primary care, inpatients, outpatients, pharmacies and etc.) that are needed to inform targeted policy development and implementation.^9^ One approach for collecting community antibiotic use data is to conduct household surveys, but these are costly, time consuming and therefore not conducted regularly at a country level.^10,11^ Hospital electronic pharmacy databases record longitudinal antibiotic use in the hospital and, when combined with hospital admission data, can be used to analyse aggregated inpatient antibiotic use.^12,13^ However, such databases are challenging to extract or are unavailable in most low and middle-income countries and many high-income countries.

Disparities in data collection methods and sources pose a challenge in combining different data resources for benchmarking usage patterns across inpatient care, outpatient services and community pharmacies. Moreover, the data gap highlights the need for simple tools to estimate antibiotic use across different healthcare sectors to support policy development, implementation and evaluation of outcomes. The objective of this study was to estimate the proportion of oral antibiotic use across different healthcare sectors. We proposed a new approach of using formulation (oral/parenteral) as a proxy for whether the antibiotic was used in the community or hospital healthcare sector, when only aggregated antibiotic sales data without sector-specific usage information was available.

## Materials and methods

### Data sources

We obtained antibiotic data from eight countries where data was available from both the IQVIA MIDAS antibiotic sales data and from the Global-PPS antibiotic use data in 2019. The year 2019 was chosen because it was pre-COVID-19. These countries were chosen based on having at least two hospitals in the Global-PPS data collection in 2019. The eight countries from four of the six WHO regions were Belgium, Canada, China, the Netherlands, the Philippines, Saudi Arabia, Singapore and the UK. IQVIA collected data on national estimated antibiotic sales, which included antibiotic formulations and sector (‘hospital’ versus ‘retail’) from which the data were from, whereas the Global-PPS collected patient-level data on antimicrobial prescribing in hospital facilities.

The hospital antibiotic prescribing data were collected using a 1-day point-prevalence survey on inpatient hospital wards according to the 2019 Global-PPS protocol. Participation in the Global-PPS project by hospitals was voluntary, and local ethical approval was obtained where necessary. All data were pseudonymized at the patient level. The Global-PPS data collection methods have been described in detail elsewhere.^4^ For each antibiotic prescription, the prescribed dose on the day of the PPS in defined daily doses (DDD) was calculated on the basis of dosing and frequency information and the WHO ATC/DDD list.^14^ The following antibiotics were included: antibacterials for systemic use (ATC J01), nitroimidazole derivatives (ATC P01AB) and antibiotics used as intestinal anti-infectives (ATC A07AA). Since the number of hospitals participating in the PPS varied across countries, the DDD was standardized to represent the DDD per 100 surveyed patients per day. Antibiotic prescriptions for children aged under 18 were excluded as currently there is no standard DDD methodology for children.

### Conversion to WHO’s defined daily dose in IQVIA MIDAS antibiotic sales data

We used IQVIA’s New Form Code Classification Guidelines version 2025 to convert antibiotic units and to classify formulations into oral (e.g. tablets, capsules or suspensions) or parenteral (e.g. ampoules, vials) routes of administration in the IQVIA MIDAS antibiotic sale data. In the original raw data, antibiotic use for a given country and year was presented in kg. DDD is defined as the assumed average maintenance dose per day for adults for its main indication and was obtained from the ATC/DDD Index website maintained by the Norwegian Institute of Public Health (a WHO Collaborating Centre). We used the kilogram weight divided by the WHO’s unit DDD to calculate antimicrobial use (AMU) in units of DDD. A few antibiotics in the IQVIA MIDAS data did not have a WHO DDD available. For these drugs, we undertook an internet search for recommended daily doses from reputable sources such as National Institute for Health and Care Excellence or the manufacturer. By these combined means, we were able to calculate AMU for 97.4% (10 829 out of 11 120 total records of antibiotic regimens) of oral antibiotics and 97.3% (5421 out of 5572 total records of antibiotic regimens) of the parenteral antibiotics in the IQVIA MIDAS data available to us. The IQVIA MIDAS data did not distinguish between antibiotic sales for children and adults. We estimated DDDs per 1000 inhabitants per day (DIDs) per country, using annual population estimates from 2019 World Bank country population.^15^

### Definitions

Antibiotics were categorized as Access, Watch or Reserve according to the 2023 WHO AWaRe classification.^9^ Our analysis considered only Access and Watch antibiotics as Reserve, not-recommended and unclassified antibiotics are of very low volumes and predominantly used in the hospital setting for complex, difficult-to-treat infections.

We will use the term ‘community healthcare sectors’ from here on to refer to antibiotic use outside of the inpatient healthcare sector. Broadly, the community healthcare sectors included emergency and ambulatory care in hospitals and clinics, primary care, local pharmacies, care homes and other sectors. The term ‘hospital healthcare sector’ refers to care provided to inpatients in health care facilities, including general and district hospitals. The definitions aligns with GLASS methodology for surveillance of national antimicrobial consumption.^16^

We defined the ‘Retail sector’ as all sectors that are outside of the hospital (that is, excluding both outpatient (e.g. in clinics) and inpatient antibiotic use in hospitals) in the IQVIA MIDAS data.

### Calculation procedure to estimate oral antibiotic use across different sector

The observed ratio of oral-to-parenteral antibiotics in the Global-PPS hospital healthcare dataset was determined for each country. A Bayesian multilevel model, assuming random country-level intercepts, was used with hospital-level aggregated hospital healthcare antibiotic use data to estimate the 95% credible interval (CrI) around the estimate of the oral-to-parenteral (Text S1 available as Supplementary data at *JAC-AMR* Online). In the base case scenario, we assumed that parenteral antibiotic formulations were consumed exclusively in hospital facilities (sensitivity analyses relaxing this assumption were performed and are described in the following section).

**Figure 1.**
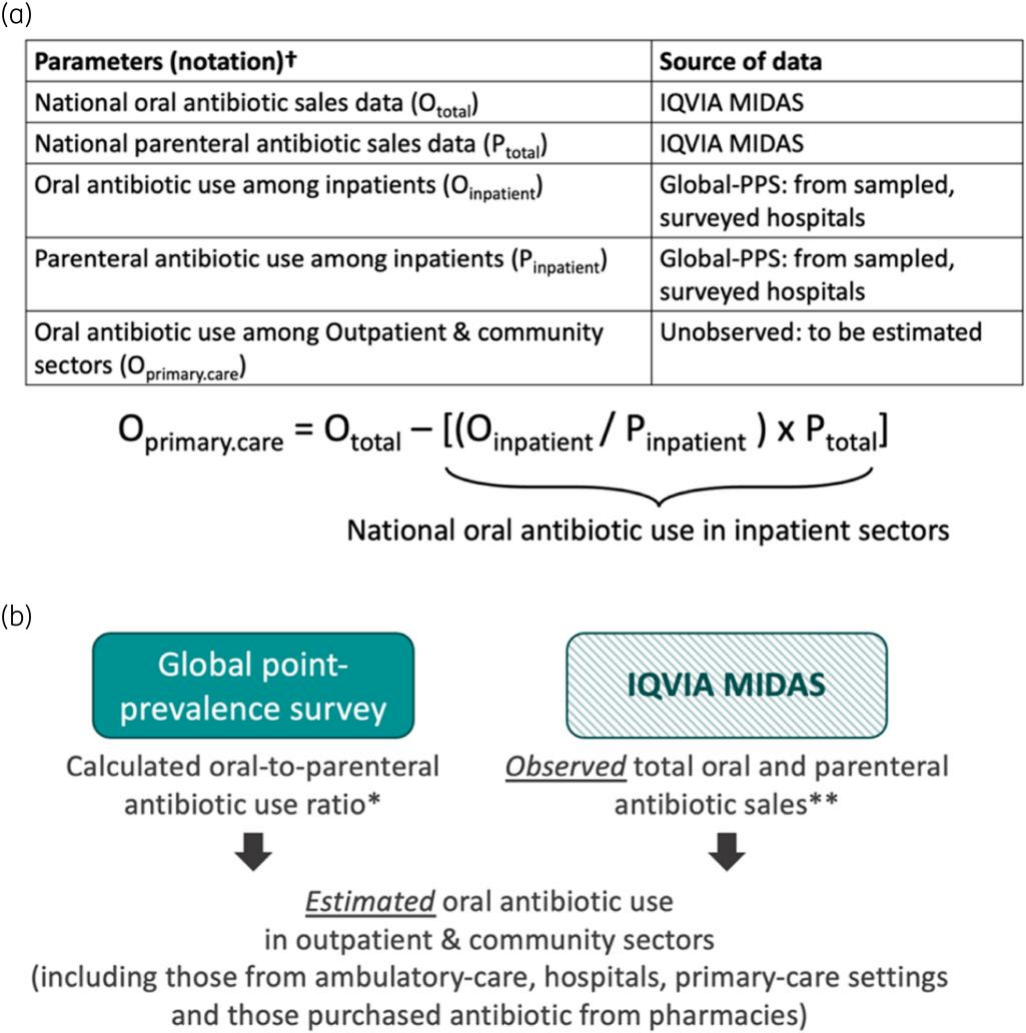
A conceptual diagram of calculation process (a) and data flow (b) to estimate antibiotic use across different sectors and validate the estimates. ^†^DDD was used in the calculation to estimate the national oral antibiotic use in hospital healthcare sector. *Global point-prevalence survey data representing hospital healthcare antibiotic use in the survey hospitals of the eight countries under analysis in 2019 was used. **Sales data were used by the authors to represent the overall country-level antibiotic use in the eight countries under analysis from the following source: IQVIA MIDAS Quarterly Sales data for 2019, reflecting estimates of real-world activity. Copyright IQVIA. All rights reserved.

Subsequently, the observed ratio of oral-to-parenteral antibiotics per country was multiplied by the total parenteral antibiotic in the IQVIA MIDAS sales data to estimate national oral antibiotic use in the community healthcare sectors (Figure 1). Data analysis was performed using R software version 4.3.2.^17^

### Validating the final estimates against antibiotic sales data from the retail sector

To validate the calculated estimates of national oral antibiotic use across hospital healthcare, and community healthcare sectors, we extracted data on oral antibiotic sales from the retail sector in the IQVIA MIDAS dataset. We then calculated the discrepancies between the estimated community healthcare sector oral antibiotic use and the observed sales data from the retail sector. We expected to see smaller differences between the estimated national oral antibiotic and the observed oral antibiotic sales from the retail sector in the IQVIA MIDAS dataset in countries with relatively strong regulations on community antibiotic use, such as Belgium, the Netherlands, Canada, Singapore and the UK, compared with countries with multiple alternative channels of antibiotic purchasing, including over-the-counter sales without a prescription.

### Sensitivity analyses

In addition, we conducted two sensitivity analyses to assess the robustness of our findings to the calculated hospital healthcare oral-to-parenteral ratios. In this set of sensitivity analyses, we assumed the oral-to-parenteral ratios were 50% lower and 50% higher, respectively, than observed in the Global-PPS 2019 data. Moreover, two sensitivity analyses to assess the robustness of the findings to the assumption that parenteral antibiotics were solely used in the hospital healthcare sectors were performed. These two analyses assumed (i) 90% and (ii) 50% of the national parenteral antibiotics sales were used in the hospital healthcare sector. The 90% assumption probably reflects typical hospital healthcare parenteral antibiotic use, whereas the 50% assumption was used to assess the robustness of the findings under extreme scenarios.

## Results

### Antibiotic use in the eight countries

The total antibiotic sales in 2019 across the eight countries was 5.59 billion DDDs (9.1 DIDs) in the IQVIA MIDAS antibiotic data. Of those antibiotics, 65% (3.49 billion DDDs) were included in the 2023 WHO Essential Medicine List.^18^ The total Access and Watch antibiotic use was 5.36 billion DDDs (96% of total use). The observed total oral antibiotics use ranged from 4.95 DIDs in the Philippines to 24.27 DIDs in Belgium (Table S2).

The 2019 Global-PPS study data from the eight countries reported 16 351 DDDs (54.4 DDDs per 100 surveyed adult patients on the day of the PPS) of Access and Watch antibiotics (Table 1). There were variations in antibiotic choice across the eight countries (Figure S1). For example, amoxicillin/clavulanic acid ranked among the top three most commonly prescribed Access antibiotics in surveyed hospitals for inpatients in Belgium, the Netherlands and Singapore, but not in the other five countries.

**Table 1.** Observed hospital healthcare antibiotic use from the 2019 Global Point Prevalence Survey study data of eight countries

		Observed absolute antibiotic use measured in number of DDD prescribed/100 surveyed adults on the day of the PPS		
Countries^a^	AWaRe category	Oral antibiotics	Parenteral antibiotics	Estimates of oral-to-parenteral antibiotic ratios (95% CrI)
Belgium	Access	8.3	18.4	0.49 (0.40–0.61)
Belgium	Watch	5.6	10.6	0.55 (0.46–0.65)
Canada	Access	8.0	12.8	0.77 (0.51–1.19)
Canada	Watch	6.3	17.1	0.42 (0.30–0.58)
China	Access	4.3	10.8	0.53 (0.25–1.09)
China	Watch	2.4	58.3	0.05 (0.03–0.09)
Netherlands	Access	9.3	22.1	0.47 (0.19–1.05)
Netherlands	Watch	7.4	14.7	0.34 (0.15–0.79)
Philippines	Access	9.0	12.5	0.58 (0.43–0.77)
Philippines	Watch	25.9	30.3	0.84 (0.67–1.05)
Saudi Arabia	Access	5.4	26.2	0.23 (0.12–0.49)
Saudi Arabia	Watch	6.6	51.8	0.16 (0.10–0.27)
Singapore	Access	13.0	18.5	0.62 (0.31–1.22)
Singapore	Watch	7.5	16.5	0.41 (0.23–0.73)
UK	Access	17.6	23.2	1.01 (0.56–1.80)
UK	Watch	8.7	14.9	0.57 (0.37–0.89)

^a^The number of hospitals participating in Global-PPS in 2019 was 56, 14, 4, 2, 31, 6, 4 and 8 in Belgium, Canada, China, Netherlands, Philippines, Saudi Arabia, Singapore and the UK, respectively (details in Table S1).

### Oral-to-parenteral antibiotic use ratio in Global-PPS data

Across all eight countries, the ratio of oral-to-parenteral antibiotic use in the Global-PPS data was below the value of 1, except for Access antibiotics in the UK [1.01 (95%CrI: 0.56–1.80)], implying oral and parenteral antibiotic usage was similar among the hospital healthcare population (Table 1 and Figures S2 and S3). The ratios of oral-to-parenteral antibiotic use were similar across three European countries. Small oral-to-parenteral ratios were observed for Watch antibiotics in China and for Access and Watch antibiotics in Saudi Arabia, suggesting there were large variation in the relative volumes of oral and parenteral antibiotics used in the hospital inpatient population.

### Estimated proportion of oral antibiotic usage in hospital healthcare settings

After applying the ratios derived from the Global-PPS data to the IQVIA MIDAS data, estimates indicated that the proportion of oral antibiotics were used by hospital healthcare ranged from 0.3% (95%CrI: 0.2%–0.6%) to 6.3% (95%CrI: 3.0%–13.1%) across the eight countries analysed (Table 2; Table S3 and S4). When testing different oral-to-parenteral ratios, even with a ratio 1.5 times the observed value, >94% of oral antibiotics were still used in non-hospital healthcare sectors for most countries, except China. Similarly, when examining the assumption that parenteral antibiotics were predominantly used in hospital healthcare settings, even if 50% would be used in non-hospital healthcare settings, the estimated proportion of parenteral Access and Watch antibiotics used in hospital healthcare sectors remained above 90% in most of the countries included in the analysis (Table S4).

**Table 2.** The overall observed national oral Access and Watch antibiotic use compared with the estimated oral antibiotic use within hospital healthcare sectors

AWaRe group	Country	Total observed national oral antibiotic use in millions DDD^a^	Estimated national oral antibiotic use in hospital healthcare sector in millions DDD (95% CrI)	Estimated percentage of oral antibiotic use by inpatients in the hospital healthcare sectors out of the total national oral antibiotic use (95% CrI)
Access	Belgium	63.51	1.42 (1.14–1.76)	2.2% (1.8–2.8%)
Access	Canada	144.05	2.42 (1.61–3.73)	1.7% (1.1–2.6%)
Access	China	1185.56	75.26 (35.48–155.59)	6.3% (3.0–13.1%)
Access	Netherlands	42.12	0.78 (0.32–1.75)	1.9% (0.8–4.2%)
Access	Philippines	97.95	0.78 (0.58–1.04)	0.8% (0.6–1.1%)
Access	Saudi Arabia	138.36	0.43 (0.21–0.88)	0.3% (0.2–0.6%)
Access	Singapore	13.39	0.39 (0.20–0.78)	2.9% (1.5–5.8%)
Access	UK	309.61	11.80 (6.59–21.05)	3.8% (2.1–6.8%)
Watch	Belgium	38.18	1.13 (0.95–1.33)	3.0% (2.5–3.5%)
Watch	Canada	61.92	2.25 (1.61–3.13)	3.6% (2.6–5.0%)
Watch	China	2153.73	22.35 (12.05–42.77)	1.0% (0.6–2.0%)
Watch	Netherlands	15.92	0.42 (0.18–0.98)	2.7% (1.2–6.2%)
Watch	Philippines	97.37	3.04 (2.42–3.80)	3.1% (2.5–3.9%)
Watch	Saudi Arabia	123.59	1.23 (0.75–2.03)	1.0% (0.6–1.6%)
Watch	Singapore	8.12	0.22 (0.12–0.39)	2.7% (1.5–4.8%)
Watch	UK	146.32	5.39 (3.47–8.30)	3.7% (2.4–5.7%)

^a^A summary of data from the following IQVIA information service. Source: IQVIA MIDAS Quarterly Sales data for 2019, reflecting estimates of real-world activity. Copyright IQVIA. All rights reserved.

### Differences between estimated oral antibiotics and observed oral antibiotics in retail sectors

We used the data on oral antibiotic sales from the retail sector in the IQVIA MIDAS dataset to validate the estimated oral antibiotic use across hospital healthcare, and community healthcare sectors. The estimated oral antibiotic use outside of the hospital healthcare sectors was larger than the observed oral antibiotics from the retail sector [all sectors that are outside of the hospital that is, excluding both outpatient (e.g. in clinics) and inpatient antibiotic use in hospitals] in all countries except for Singapore (Table 3). This could be due to multiple channels of antibiotic access, including retail sectors and outpatient care in hospitals and/or overstock within the community and primary health care sector. The differences between the estimated oral antibiotic use of community healthcare sectors and the observed oral antibiotic purchased by the retail sector were <2 DID, except in Saudi Arabia. This confirmed the expectation that most of the primary health care antibiotics were purchased through the retail sectors in high-income settings where community antibiotic use is generally well-regulated. In all high-income settings, the estimated oral Access antibiotic use was higher than the estimated oral Watch antibiotics in the primary health care sectors. In China and the Philippines, higher oral Watch antibiotic was observed.

**Table 3.** Variation between the estimated national oral antibiotic use among community healthcare sector and observed oral antibiotics purchased by retail sectors

AWaRe	Country	Estimated national oral antibiotic use in the community healthcare sectors in millions DDD (95% CrI)	Observed oral antibiotics purchased by retail sector in the IQVIA MIDAS 2019 data in millions DDD	Absolute difference in millions DDD^a^	Absolute difference in DID^b^
Access	Belgium	62.10 (61.75–62.38)	60.352	1.744	0.416
Access	Canada	141.63 (140.32–142.44)	138.748	2.880	0.210
Access	China	1110.30 (1029.97–1150.08)	767.836	342.460	0.666
Access	Netherlands	41.34 (40.36–41.79)	40.418	0.918	0.145
Access	Philippines	97.17 (96.91–97.37)	94.698	2.474	0.063
Access	Saudi Arabia	137.94 (137.48–138.15)	110.401	27.537	2.202
Access	Singapore	13.00 (12.62–13.19)	13.014	−0.015	−0.007
Access	UK	297.81 (288.56–303.02)	256.210	41.602	1.705
Watch	Belgium	37.05 (36.85–37.23)	32.671	4.382	1.045
Watch	Canada	59.67 (58.79–60.31)	58.146	1.525	0.111
Watch	China	2131.38 (2110.97–2141.68)	1127.783	1003.597	1.953
Watch	Netherlands	15.50 (14.94–15.74)	15.135	0.364	0.057
Watch	Philippines	94.32 (93.56–94.94)	91.609	2.713	0.069
Watch	Saudi Arabia	122.36 (121.56–122.84)	91.679	30.683	2.453
Watch	Singapore	7.91 (7.73–8.00)	8.102	−0.195	−0.094
Watch	UK	140.93 (138.02–142.85)	128.733	12.199	0.500

^a^Absolute differences between point estimate of national oral antibiotic use in the community healthcare sectors and the observed oral antibiotic purchased by retail sectors.

^b^Absolute difference in DID is the absolute difference in DDD standardized by the country population size; and in 2019 the populations size of Belgium, Canada, China, Netherlands, Philippines, Saudi Arabia, Singapore and the UK were 11 488 980, 37 601 230, 1 407 745 000, 17 344 874, 108 116 622, 34 268 529, 5 703 569 and 66 836  327, respectively. Source: Based on data from the following IQVIA information service: IQVIA MIDAS Quarterly Sales data for 2019, reflecting estimates of real-world activity. Copyright IQVIA. All rights reserved

## Discussion

This study demonstrates the potential of using antibiotic administration routes as a proxy to estimate antibiotic use across different healthcare sectors. We estimate that <7% of national oral Access and Watch antibiotics procurement was used in the hospital healthcare sector. Our finding supports the expectation that most oral antibiotics are used outside of the hospital healthcare sector in the selected countries.^19–21^ We observed variations in oral-to-parenteral use ratios among inpatients across the eight participating hospitals in the Global-PPS 2019 data. Among the Access antibiotics, the oral-to-parenteral ratio ranged from 0.23 in Saudi Arabia, suggesting substantially lower oral use compared with parenteral antibiotic use, to 1.01 in the UK, suggesting similar oral and parenteral Access antibiotic use. These variations are probably due to differences in antibiotic use patterns between countries. In the UK, oral amoxicillin (± clavulanic acid) was the most widely used antibiotic (Figure S1) in the Global-PPS 2019 data, and its use was higher than the parenteral preparation of the same drug. It is not possible to comprehensively assess the reasons for higher oral antibiotic use compared with parenteral use without longitudinal antibiotic use and clinical data. However, one of the plausible reasons would be the implementation of the UK National Institute for Health and Care Excellence antimicrobial stewardship guidelines on switching from intravenous to oral antibiotics.^22^ Rapid early parenteral to oral switching accompanied by a high hospital discharge rate could contribute to the observed high oral antibiotic use.^23^ Our observed variations in antibiotic use patterns across different countries could also be due to differences in healthcare provision, antimicrobial stewardship programmes, differences in infectious disease epidemiology,^24^ causative pathogen burdens in hospitals across different settings,^1^ availability of parenteral and oral formulations of different antibiotics, and clinical characteristics of patients. This further highlights the importance of collecting local data to support evidence-based guidelines for targeted antimicrobial stewardship programmes.

We observed less variation in oral-to-parenteral antibiotic use ratios in the Watch antibiotics. All countries had ratios <1 suggesting higher parenteral compared with oral Watch antibiotic use. Watch antibiotics are often used for patients with severe infections who may need high dose intravenous antibiotics or may not tolerate oral medicines. Nevertheless, overuse of parenteral Watch antibiotics is also likely in many settings.^25^ Moreover, some Watch antibiotics such as ceftriaxone, commonly used for suspected sepsis, only have parenteral formulations and were among the top 90% of Watch antibiotics used in most hospitals in the Global-PPS 2019 (Figure S1). The oral-to-parenteral ratio of Watch antibiotics in the Philippines was large, and the upper bound of the uncertainty interval was above 1 [oral-to-parenteral ratio: 0.84 (95%CrI: 0.67–1.05)]. One reason for this observation is the high oral cefuroxime use for surgical prophylaxis in the 31 participating hospitals in 2019. In the Philippines, a high rate of surgical prophylaxis prescriptions was noted, especially in Obstetrics and Gynaecology departments. Previous studies have shown low compliance with guidelines on antibiotic choice, route and duration in surgery, with prolonged use being common.^26,27^

The estimated low proportion of national oral Access and Watch antibiotics that were used in the hospital healthcare sectors was robust against a wide range of calculated hospital healthcare oral-to-parenteral antibiotic ratio values (Table S3). For instance, even when assuming a high hospital healthcare oral-to-parenteral ratio of 1.5 times the observed ratio, most countries would still be expected to have ∼95% of national oral antibiotics used in the non-hospital healthcare sectors. One exception was China, which had a low overall proportion of oral antibiotic procurement (Table S2). This aligns with a previous study from China, which reported oral antibiotics accounted for 64% of national antibiotic use.^28^ Nevertheless, the overall observed oral (6.5 DID) and parenteral (1.4 DID) antibiotic sales in IQVIA MIDAS 2019 data was lower than reported by Yang *et al.* (oral and parenteral antibiotic usage was 9.30 DID and 5.15 DID, respectively) from data of public health institution and during COVID-19.^28^

Overall, the findings from our study are consistent with the WHO GLASS-AMU’s suggestion that oral antibiotic use can be a surrogate for use in community and primary care.^7^ However, more granular data on antibiotic use from different health sectors are needed to inform targeted antimicrobial stewardship programmes. To achieve the target of at least 70% of human antibiotic use being Access antibiotics,^3,29^ our findings suggest that reducing national and global oral Watch antibiotic use would be essential. The volume of Watch oral antibiotic procurement was higher than Access oral antibiotic procurement in China, the Philippines and Saudi Arabia (Table 2), and the estimated percentage of Watch antibiotic usage by the hospital healthcare sector was low.

There are several key limitations to this study. First, the main analysis assumes all parenteral antibiotic use occurs in hospital healthcare settings. While this assumption holds in some settings such as Lebanon, where 99% of antibiotic use in the community is represented by oral formulations,^30^ outpatient parenteral antibiotic use exists in the USA (since 1974)^31^ and other countries (including Australia, New Zealand, Canada, Singapore and the UK).^32–34^ Our sensitivity analyses suggest that even in settings where 50% of parenteral antibiotics are used in non-hospital healthcare sectors, the estimated proportion of parenteral Access and Watch antibiotics used in hospital healthcare would still remain above 90% in most of the countries included in the analysis (Table S4). However, future studies with detailed hospital antibiotic use data would be needed to systematically assess the ratio of oral-to-parenteral antibiotic use in hospitals of different health systems, particularly with varying levels of Out Patient Antibiotic Use (OPAT) services. Second, our analysis only included two low- and middle-income countries, limiting generalisability to others. In settings such as China, with a high overall proportion of parenteral antibiotic use, the proportion of Access antibiotics used in community healthcare sectors would be lower than 95% (Table 2). Future studies that cover a broader range of geographical regions, including the African and South American regions, are essential for validating the estimates generated in this study. Third, our study assumes a stable hospital healthcare oral-to-parenteral ratio over time. This assumption may not be true in certain settings, for example due to seasonal infectious disease burdens. Moreover, the coverage of hospitals participating in the Global-PPS 2019 study varies across the eight countries included in the analysis (Table S1), which affects the generalisability of the estimated oral-to-parenteral ratios for the country-level inpatient population. For instance, data from only two hospitals in the Netherlands—one with 188 adult ward beds and the other with 474 beds—were included in the estimation of oral-to-parenteral ratios. This limitation highlights the importance of a future study to explore efficient strategies for a representative sampling frame for antibiotic use point-prevalence surveys across both primary care and hospital settings. Moreover, in this proof-of-concept study, we only used data from the adult hospital healthcare sector samples of the Global-PPS 2019 study to estimate the oral-to-parenteral antibiotic use ratio, which does not sufficiently represent the paediatric antibiotic use pattern. This ratio was applied to the IQVIA MIDAS data on the overall national-level antibiotic procurement for the whole population including adults and paediatrics given the IQVIA MIDAS sales data do not allow us to differentiate between paediatric and adult usage. However, it is expected that adult use constitutes the majority. Nonetheless, standardizing the absolute measurement of antibiotic use in paediatric population is crucial to quantify and monitor national and global antibiotic use. Furthermore, our analysis does not reflect the volume of appropriate antibiotic use and may not capture antibiotic use purchased from informal or unlicensed sources. Finally, we observed variations in the oral-to-parenteral antibiotic use ratio across countries. Therefore, directly applying these ratios from one country to another should be avoided. Future analyses to validate the application of our approach to estimate national antibiotic use across healthcare sectors for settings with different antibiotic market structure and healthcare-seeking behaviours would be important. Moreover, establishing consensus on the definitions of healthcare sectors—including inpatient, outpatient, community, retail and emergency and ambulatory care—across different databases could facilitate the integration of multiple data sources in future studies.

### Conclusion

Our results indicate that where patient-level data are unavailable, alternative sources, such as antibiotic procurement and supply data that include routes of administration, can be used to estimate sector-specific national use. However, variations in antibiotic use patterns across different countries are large and innovative studies to collect antibiotic use data efficiently across different healthcare sectors are needed to support the development of locally tailored policies and interventions, and to validate our results. For instance, patterns of antibiotic use identified from macro-level import and other data could be validated using novel primary-care point-prevalence survey methods (Cook *et al.*^35^ and Boven *et al.*^36^). Sustainable citizen-led initiatives to collect community antibiotic use data could also play an important role in filling data gaps. Representative sampling frames for both surveys could then be combined with other datasets to provide robust estimates of population-level patterns of use to inform national antibiotic policies.

## Supplementary Material

dlag057_Supplementary_Data

## Supplementary data

Figures S1–S3 and Tables S1–S4 are available as Supplementary data at *JAC-AMR* Online.
