# Supplementary material for: Using oral and parenteral formulation of AWaRe antibiotics as a proxy estimate of community and hospital healthcare sector use
Source: JAC Antimicrob Resist. 2026 Apr 30;8(3):dlag057. doi: 10.1093/jacamr/dlag057 (PMC13132163; doi:10.1093/jacamr/dlag057)

**Supplementary data**

**Text S1: Methods**

**Bayesian model with random intercept model**

To estimate the uncertainty interval around the calculated oral-to-parenteral antibiotic use ratio, we fitted a Bayesian hierarchical model without any predictor variable. The oral-to-parenteral antibiotic use ratios calculated from data of each participating hospital were log-transformed to avoid taking the value 0. We define the model as follows. Let *Y_ij_* denote the oral-to-parenteral antibiotic use ratio on a log scale for hospital *i* in country *j*. The form of our model is

Y*_ij_* ~ *N(μ_ij_ , σ_j_^2^)*

*μ_ij_* = β_0_*_j_*

We assumed a vague prior for β that is normally distributed with mean 0 and a large variance (value of 5^2^) to represent a weakly informed prior. We assumed *σ* follows a Cauchy distribution with median 0 and scale parameter of 2.

In total**,** 248 oral-to-parenteral antibiotic use ratios were calculated from 125 participating hospitals in 2019 (Appendix Figure 2). For the participating hospitals without observations for a particular class of antibiotics (Access/Watch) observed on the day of the survey, we avoided assigning a value of 0 (which could result in an undefined mathematical value when calculating the ratio) by assuming that the use was minimal and assigning a value of 1 DDD. Of the 125 participating hospitals, there were 3 hospitals without an observed parenteral Access antibiotic, 2 without an observed parenteral Watch antibiotic, 2 without oral Access antibiotic, and 1 without observed oral Watch antibiotic on the day of the survey.

**Table S1.** The number of hospitals participating in Global-PPS in 2019.

| **Country** | **Number of hospitals participating in the 2019 survey** | **Median (minimum, maximum) number of beds in adult wards surveyed per participating hospital** |
| --- | --- | --- |
| Belgium | 56 | 265 (30, 1024) |
| Canada | 14 | 277.5 (34, 641) |
| China | 4 | 127.5 (108, 142) |
| Netherlands | 2 | 331 (188, 474) |
| Philippines | 31 | 247 (12, 706) |
| Saudi Arabia | 6 | 124 (20, 283) |
| Singapore | 4 | 825 (668, 1393) |
| United Kingdom | 8 | 195.5 (48, 433) |

**Table S2.** Observed combined Access and Watch antibiotic consumption from IQVIA MIDAS 2019 data of eight countries.

| **Countries included in the analysis** | ***Observed oral antibiotic wholesales measure in millions DDD (DID)** | ***Observed parenteral antibiotic wholesales measure in millions DDD (DID)** | **Proportion of observed oral antibiotics** |
| --- | --- | --- | --- |
| Belgium | 101,774,342 (24.27) | 5,010,667 (1.19) | 95.3% |
| Canada | 206,027,097 (15.01) | 8,847,642 (0.64) | 95.9% |
| China | 3,461,131,988 (6.74) | 708,353,513 (1.38) | 83.0% |
| Netherlands | 58,053,214 (9.17) | 2,915,291 (0.46) | 95.2% |
| Philippines | 195,423,103 (4.95) | 5,018,548 (0.13) | 97.5% |
| Saudi Arabia | 262,051,283 (20.95) | 9,682,879 (0.77) | 96.4% |
| Singapore | 21,599,786 (10.38) | 1,241,065 (0.60) | 94.6% |
| United Kingdom | 456,280,395 (18.70) | 22,127,364 (0.91) | 95.4% |

Footnote: DDD= defined daily dose; DID= DDD per 1,000 inhabitants. *Based on data from the following IQVIA information service: IQVIA MIDAS Quarterly Sales data for 2019, reflecting estimates of real-world activity. Copyright IQVIA. All rights reserved.

**Table S3.** Results from the two sensitivity analyses to explore the robustness of the findings against the calculated oral-to-parenteral antibiotic use ratio.

|  |  | **Sensitivity analysis 1** | | | **Sensitivity analysis 2** | | |
| --- | --- | --- | --- | --- | --- | --- | --- |
| **Country** | **AWaRe group** | **O-to-P ratio*** | **Estimated national oral antibiotic use in hospital healthcare sector in millions DDD** | **Estimated percentage of oral antibiotic use among hospital healthcare population**** | **O-to-P ratio*** | **Estimated national oral antibiotic use in hospital healthcare sector in millions DDD** | **Estimated percentage of oral antibiotic use among hospital healthcare population**** |
| Belgium | Access | 0.2266 | 0.65 | 1.0% | 0.6798 | 1.95 | 3.1% |
| Canada | Access | 0.3121 | 0.98 | 0.7% | 0.9364 | 2.94 | 2.0% |
| China | Access | 0.1994 | 28.55 | 2.4% | 0.5981 | 85.64 | 7.2% |
| Netherlands | Access | 0.2097 | 0.35 | 0.8% | 0.6290 | 1.05 | 2.5% |
| Philippines | Access | 0.3599 | 0.49 | 0.5% | 1.0797 | 1.46 | 1.5% |
| Saudi Arabia | Access | 0.1036 | 0.19 | 0.1% | 0.3109 | 0.56 | 0.4% |
| Singapore | Access | 0.3529 | 0.22 | 1.7% | 1.0588 | 0.67 | 5.0% |
| United Kingdom | Access | 0.3795 | 4.43 | 1.4% | 1.1384 | 13.29 | 4.3% |
| Belgium | Watch | 0.2608 | 0.54 | 1.4% | 0.7824 | 1.61 | 4.2% |
| Canada | Watch | 0.1840 | 0.99 | 1.6% | 0.5521 | 2.98 | 4.8% |
| China | Watch | 0.0202 | 9.47 | 0.4% | 0.0606 | 28.41 | 1.3% |
| Netherlands | Watch | 0.2527 | 0.31 | 2.0% | 0.7581 | 0.94 | 5.9% |
| Philippines | Watch | 0.4270 | 1.55 | 1.6% | 1.2811 | 4.65 | 4.8% |
| Saudi Arabia | Watch | 0.0640 | 0.48 | 0.4% | 0.1921 | 1.46 | 1.2% |
| Singapore | Watch | 0.2268 | 0.12 | 1.5% | 0.6805 | 0.36 | 4.5% |
| United Kingdom | Watch | 0.2932 | 2.75 | 1.9% | 0.8797 | 8.24 | 5.6% |

*oral-to-parenteral ratios used in the sensitivity analysis to calculate the estimated national oral antibiotic use in the hospital healthcare sector in millions DDD.

**out of the total national oral antibiotic use.

Footnote: In sensitivity analyses 1 and 2, we assumed the oral-to-parenteral ratio was fifty percent lower and fifty percent higher, respectively, than observed in the Global-PPS 2019 data. Source: Based on data from the following IQVIA information service: IQVIA MIDAS Quarterly Sales data for 2019, reflecting estimates of real-world activity. Copyright IQVIA. All rights reserved

**Table S4.** Results from the two sensitivity analyses to explore the robustness of the findings against the assumption of all parenteral antibiotics being used in the hospital healthcare sector.

|  |  | **Sensitivity analysis 3** | | | **Sensitivity analysis 4** | | |
| --- | --- | --- | --- | --- | --- | --- | --- |
| **Country** | **AWaRe group** | **Proportion of parenteral antibiotics used in the hospital healthcare sector** | **Estimated national oral antibiotic consumption in hospital healthcare sector in millions DDD** | **Estimated percentage of oral antibiotic use among hospital healthcare population**** | **Proportion of parenteral antibiotics used in the hospital healthcare sector*** | **Estimated national oral antibiotic use in hospital healthcare sector in millions DDD** | **Estimated percentage of oral antibiotic use among hospital healthcare population**** |
| Belgium | Access | 90% | 1.45 | 2.3% | 50% | 2.61 | 4.1% |
| Canada | Access | 90% | 1.82 | 1.3% | 50% | 3.27 | 2.3% |
| China | Access | 90% | 99.31 | 8.4% | 50% | 178.76 | 15.1% |
| Netherlands | Access | 90% | 0.68 | 1.6% | 50% | 1.23 | 2.9% |
| Philippines | Access | 90% | 0.60 | 0.6% | 50% | 1.08 | 1.1% |
| Saudi Arabia | Access | 90% | 0.08 | 0.1% | 50% | 0.15 | 0.1% |
| Singapore | Access | 90% | 0.30 | 2.2% | 50% | 0.53 | 4.0% |
| United Kingdom | Access | 90% | 6.55 | 2.1% | 50% | 11.80 | 3.8% |
| Belgium | Watch | 90% | 1.65 | 4.3% | 50% | 2.96 | 7.8% |
| Canada | Watch | 90% | 5.11 | 8.3% | 50% | 9.20 | 14.9% |
| China | Watch | 90% | 107.97 | 5.0% | 50% | 194.35 | 9.0% |
| Netherlands | Watch | 90% | 0.18 | 1.1% | 50% | 0.32 | 2.0% |
| Philippines | Watch | 90% | 2.84 | 2.9% | 50% | 5.12 | 5.3% |
| Saudi Arabia | Watch | 90% | 3.82 | 3.1% | 50% | 6.87 | 5.6% |
| Singapore | Watch | 90% | 0.45 | 5.5% | 50% | 0.81 | 10.0% |
| United Kingdom | Watch | 90% | 6.11 | 4.2% | 50% | 10.99 | 7.5% |

oral-to-parenteral ratio used in the sensitivity analysis to calculated the estimated national oral antibiotic use in hospital healthcare sector in millions DDD

**out of the total national oral antibiotic use.

Footnote: In sensitivity analyses 3 and 4, we assumed 90% and 50% of the national parenteral antibiotics procured were used in the hospital healthcare sector. Source: Based on data from the following IQVIA information service: IQVIA MIDAS Quarterly Sales data for 2019, reflecting estimates of real-world activity. Copyright IQVIA. All rights reserved

**Figure S1.** Distribution of different types of oral and parenteral antibiotics accounting for 90% of the prescriptions used by inpatients in hospital healthcare on the day that Global-PPS was performed in the participating hospital across the eight countries included in the analysis. The types of Access antibiotic (A and B) used varied by route of administration across the countries. For example, in China oral administration of amoxicillin or amoxicillin plus enzyme inhibitor was not reported in hospitals included in the 2019 Global-PPS survey, whereas the oral usage of both amoxicillin and amoxicillin plus enzyme inhibitor were the most widely used oral antibiotics in the other countries. Similarly, the number of different types of Watch antibiotics (C and D) varied across different countries and routes of administration. The graphs (A-D) were generated using the Global PPS 2019 data.

(A)


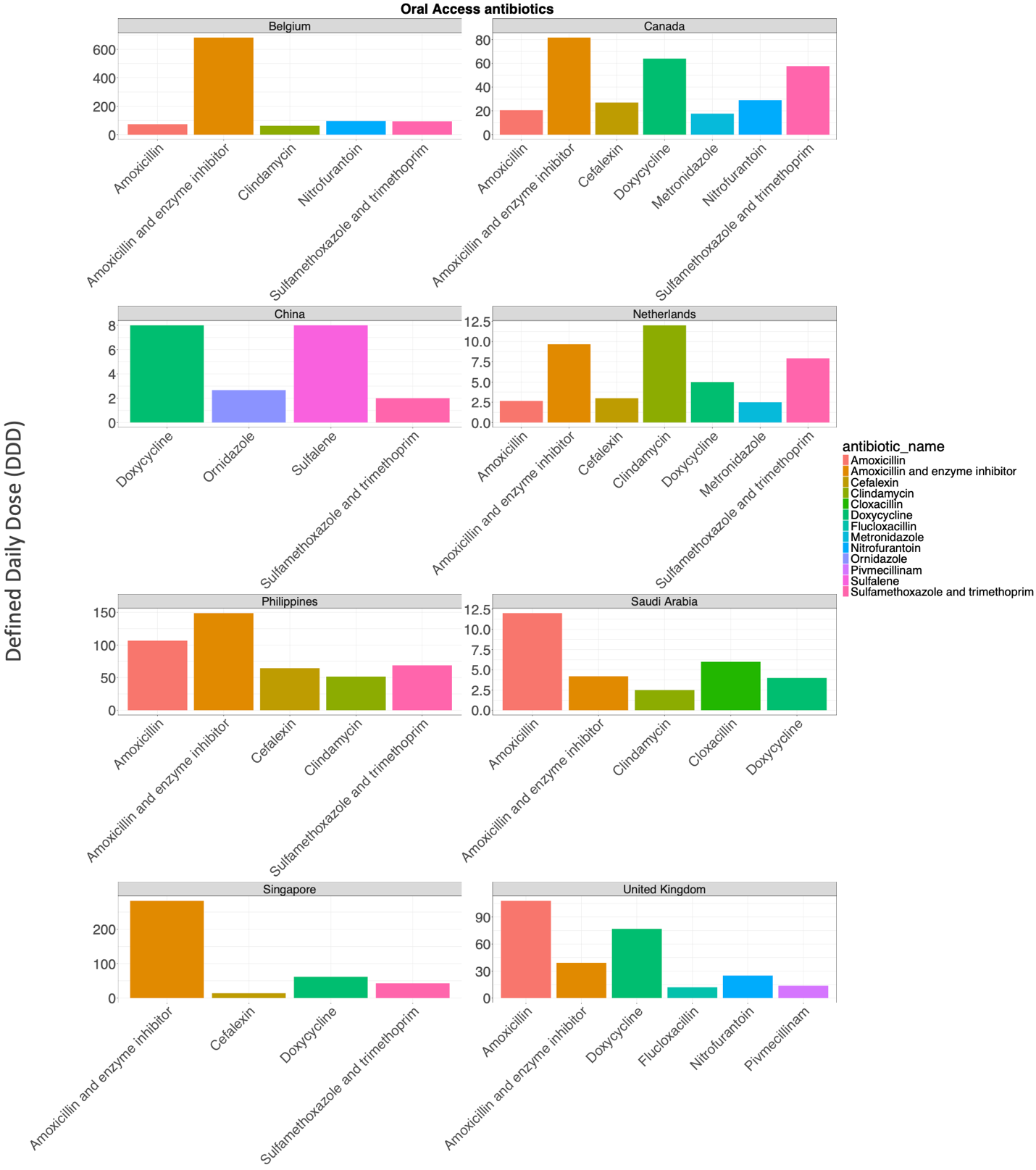


(B)


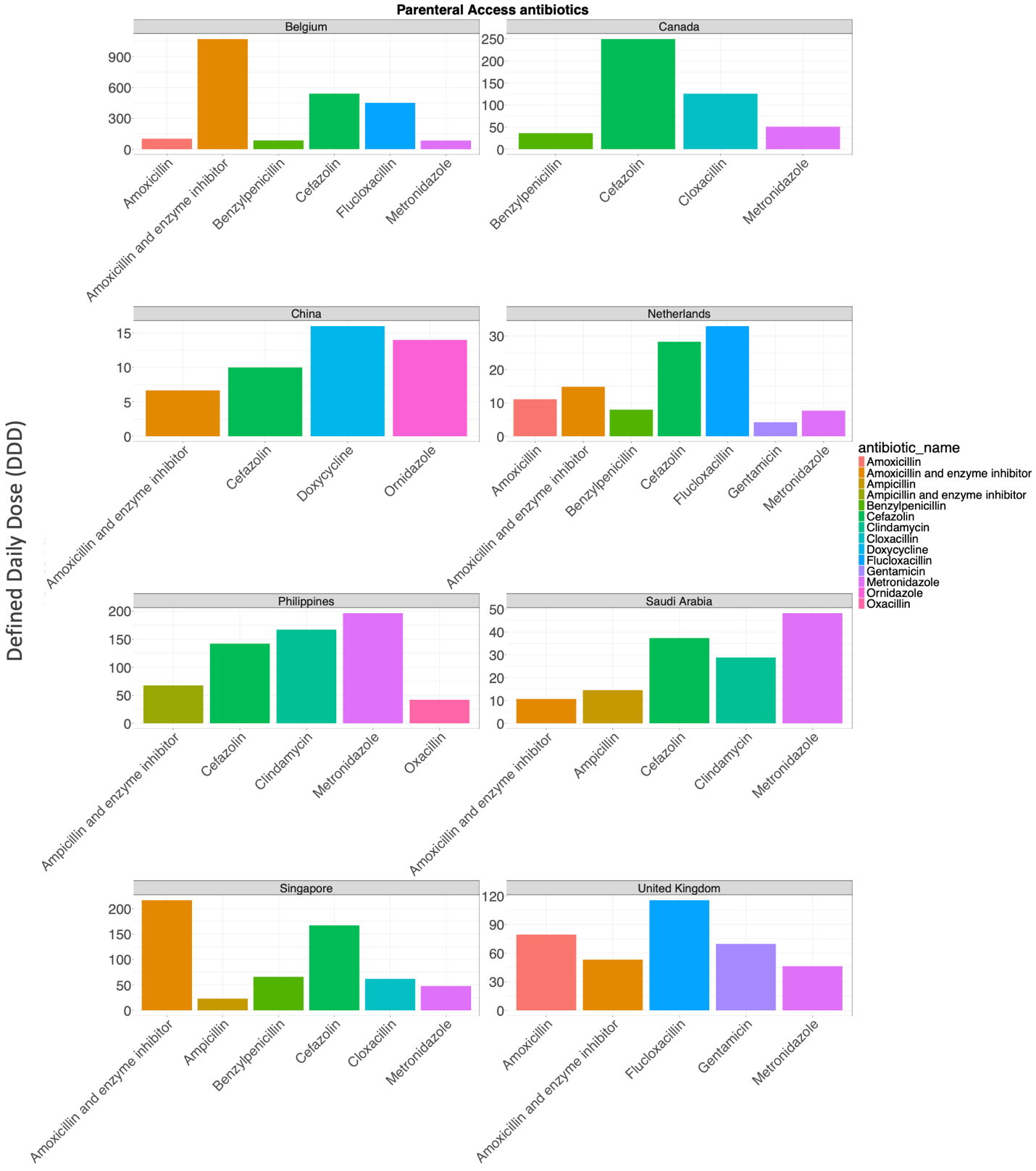


(C)
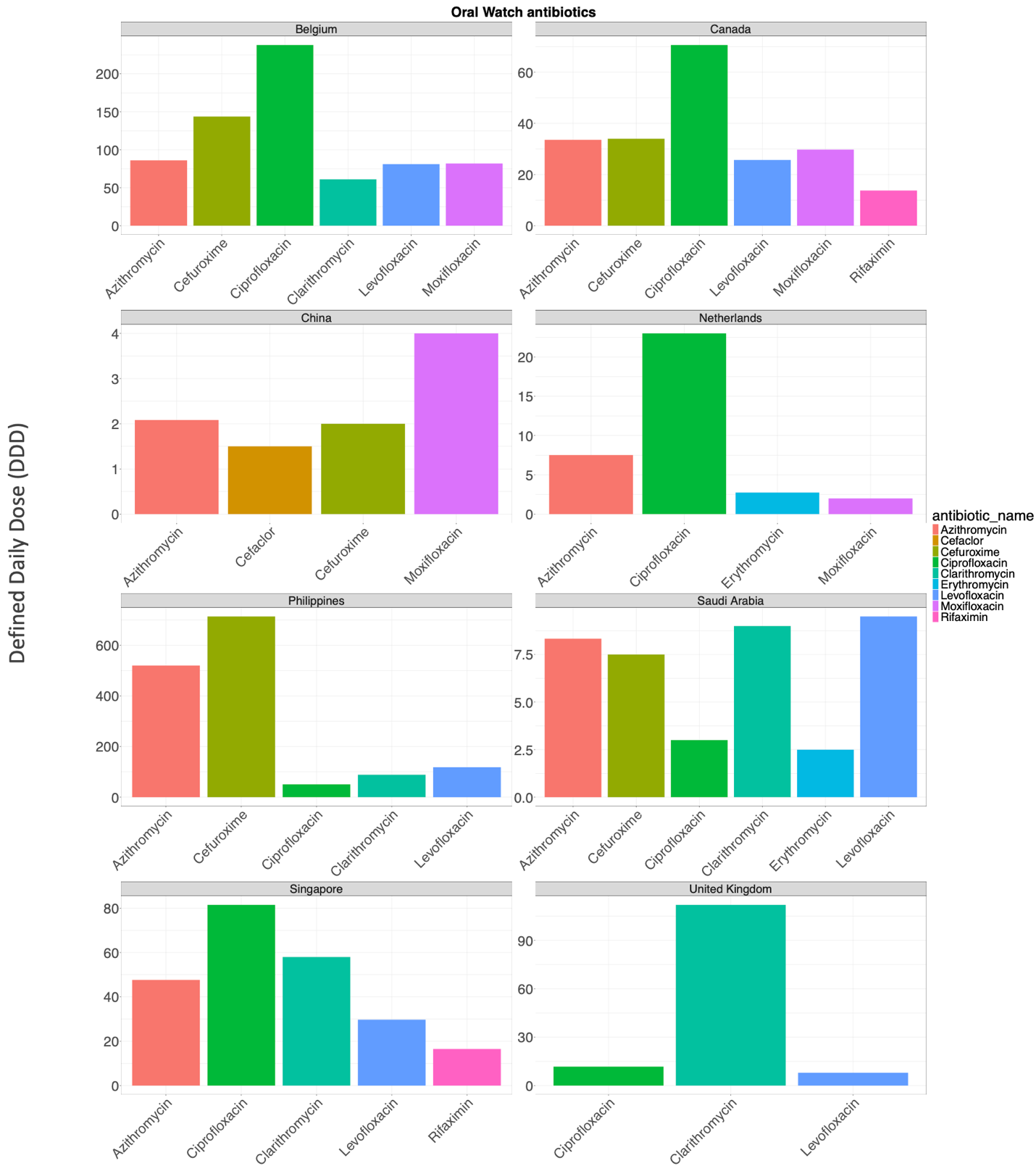


(D)
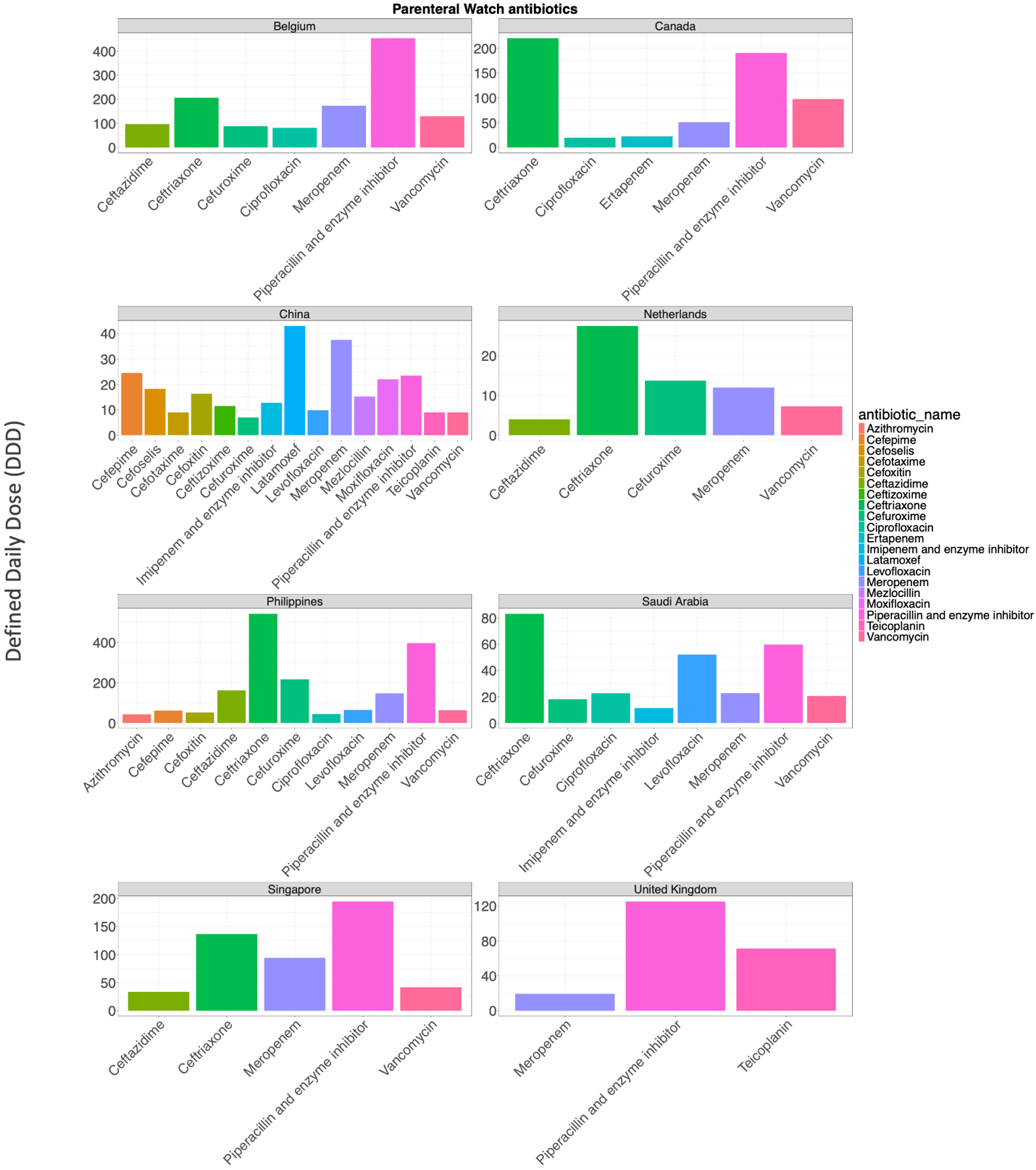


**Figure S2.** Distribution of oral-to-parenteral ratios across different surveyed hospitals by country, stratified by (A) Access and (B) Watch antibiotics.

(A)


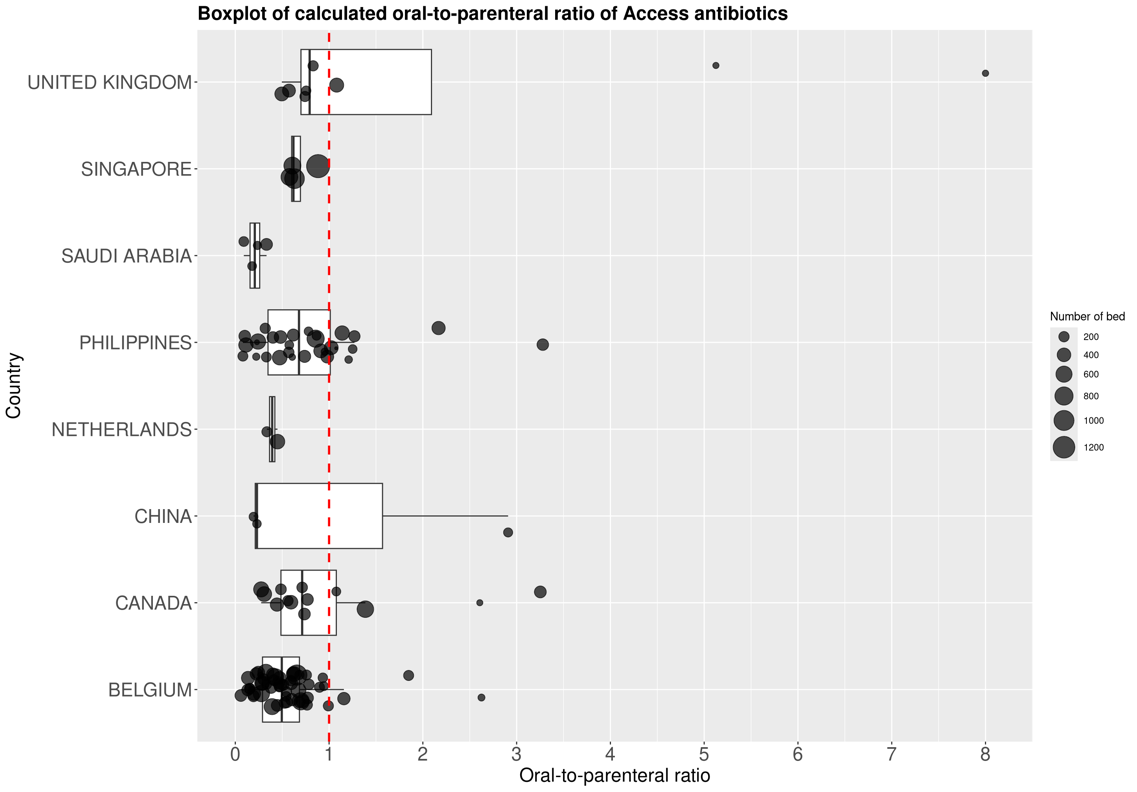


(B)


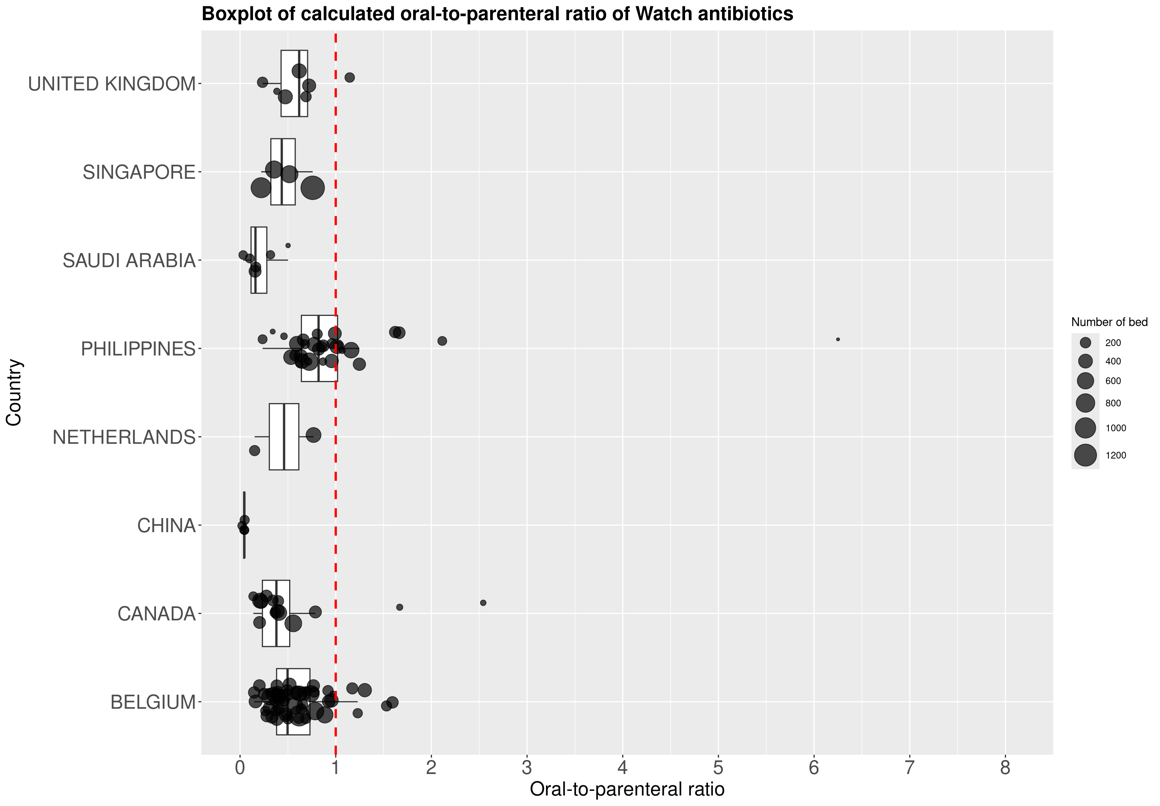


**Figure S3.** Posterior distributions from Bayesian random intercept models on log transformed oral-to-parenteral antibiotic use ratios by countries for (A) Access antibiotics and (B) Watch antibiotics.

(A)


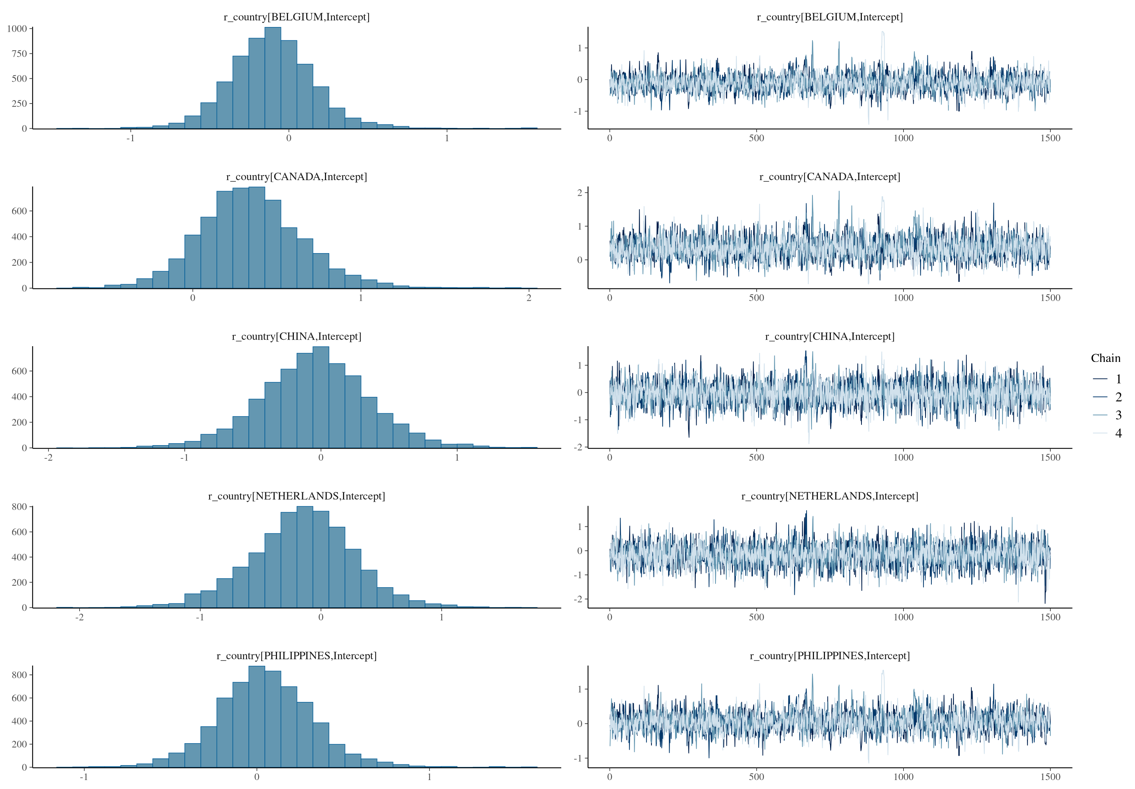


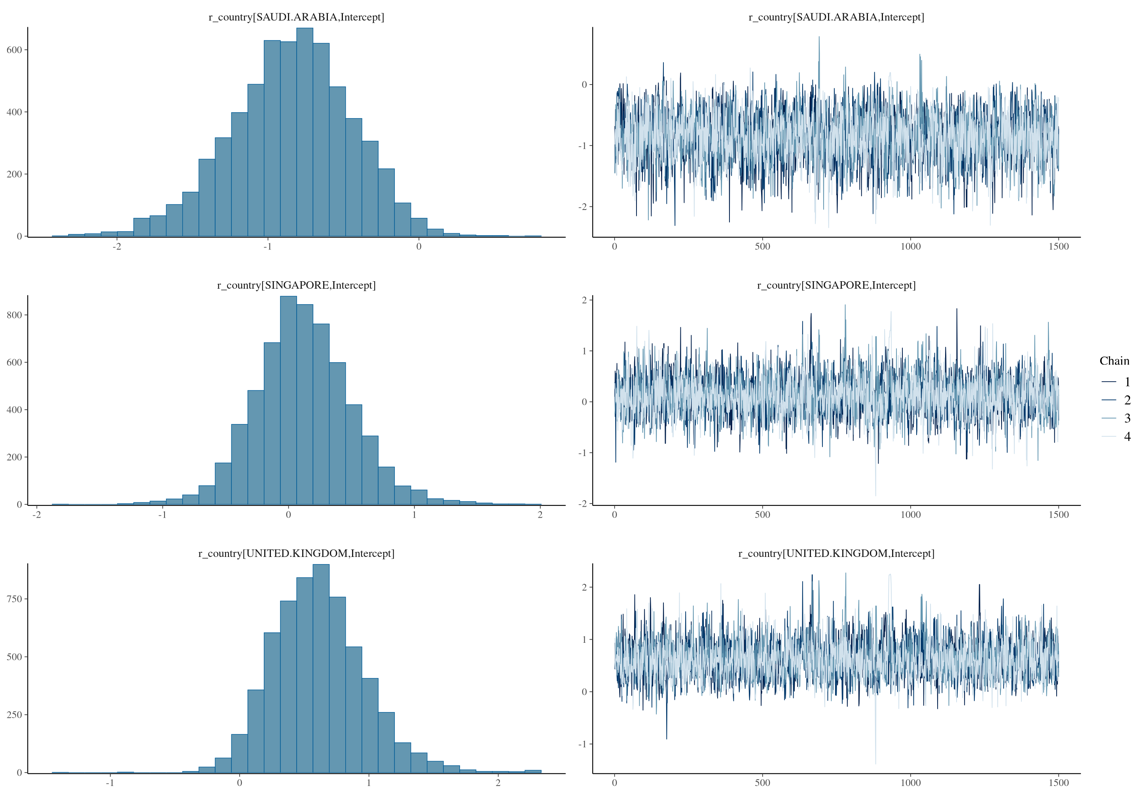


(B)


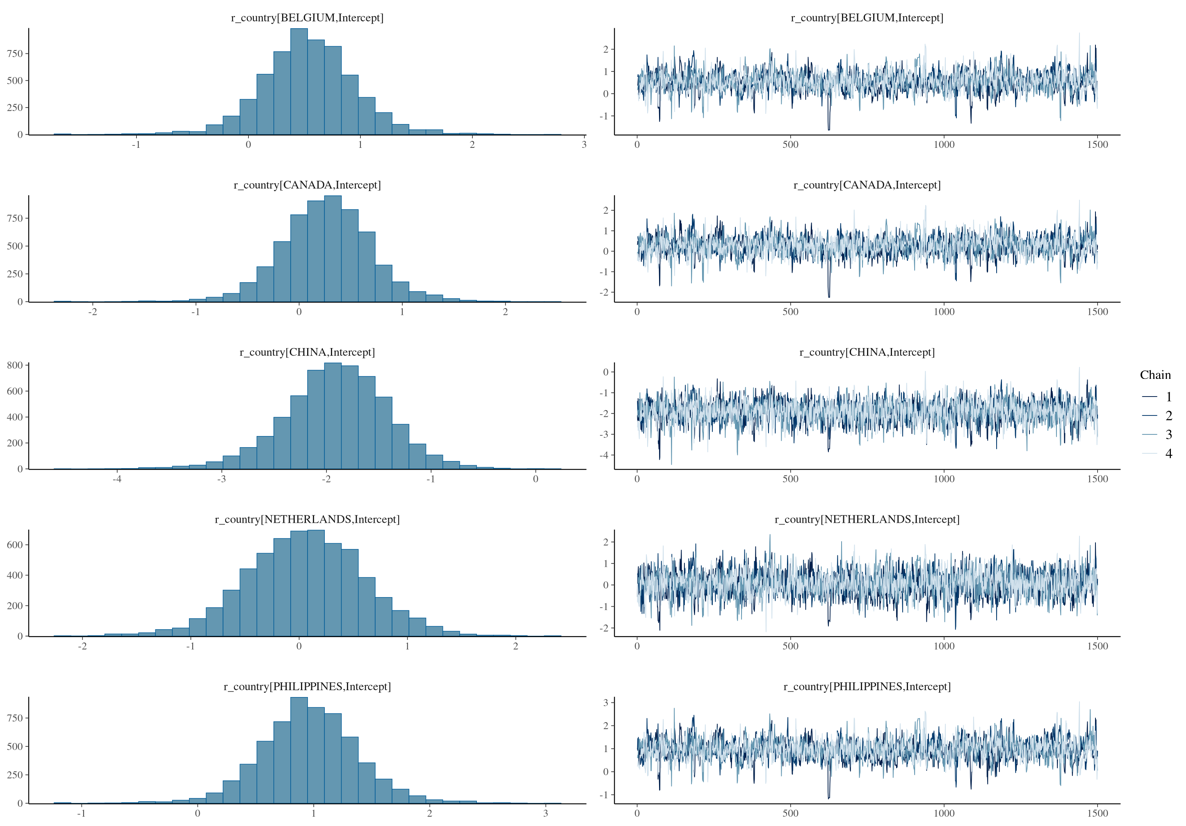


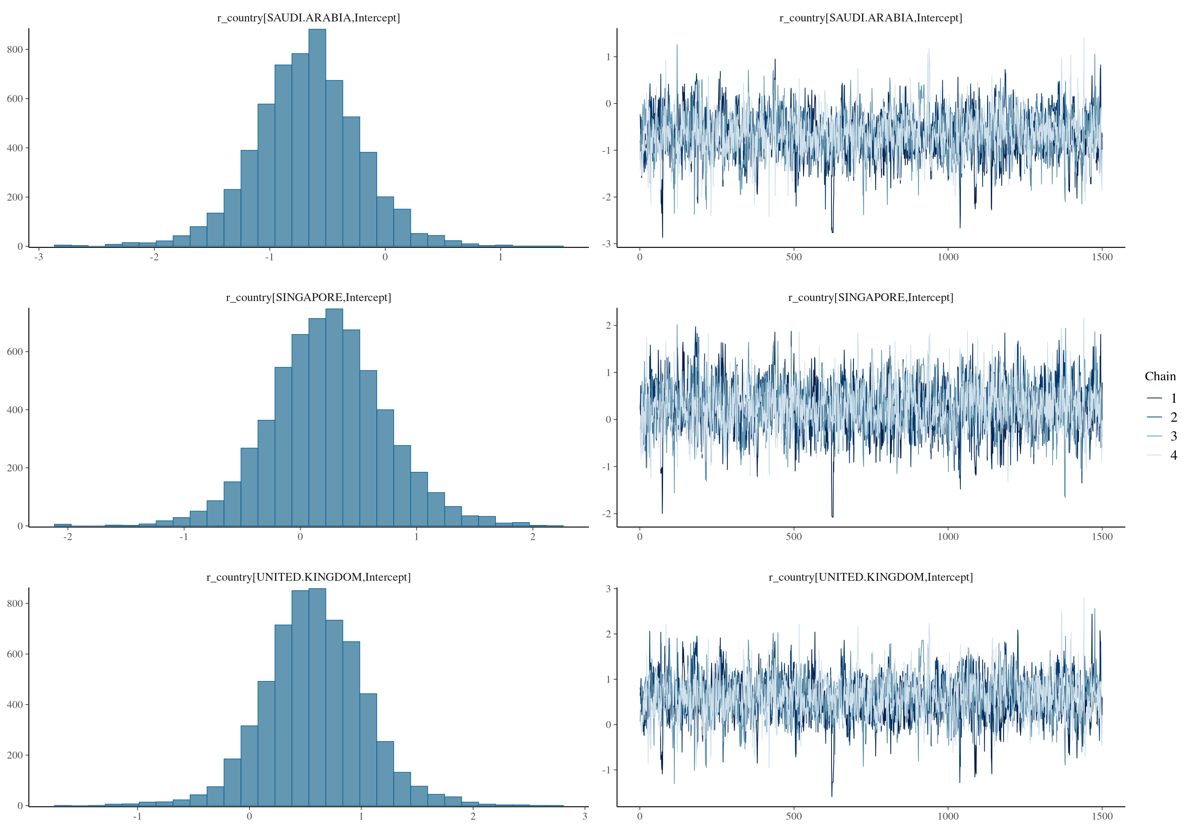

Supplement: dlag057_Supplementary_Data [file dlag057_supplementary_data.docx]
